# Candidate Faecal microRNAs as Non-Invasive Biomarkers for Bovine Paratuberculosis in *Marchigiana* Beef Cattle

**DOI:** 10.3390/ijms27125412

**Published:** 2026-06-16

**Authors:** Martina Torricelli, Laura Madeo, Anna Fratto, Andrea Felici, Linda Petrucci, Carla Sebastiani, Marco Ermini, Massimo Biagetti, Marcella Ciullo, Matteo Ricchi, Katia Cappelli, Piera Mazzone

**Affiliations:** 1 Istituto Zooprofilattico Sperimentale dell’Umbria e delle Marche “Togo Rosati” (IZSUM), 06126 Perugia, Italy; m.torricelli@izsum.it (M.T.); l.madeo@izsum.it (L.M.); a.fratto@izsum.it (A.F.); a.felici@izsum.it (A.F.); lindapetruccivet@gmail.com (L.P.); carla.sebastiani@uslumbria2.it (C.S.); m.biagetti@izsum.it (M.B.); m.ciullo@izsum.it (M.C.); 2 Azienda Sanitaria Territoriale Macerata, Servizio Veterinario di Sanità Animale, 62100 Macerata, Italy; marco.ermini@sanita.marche.it; 3 WOAH Reference Laboratory for Paratuberculosis, Istituto Zooprofilattico Sperimentale della Lombardia e dell’Emilia-Romagna “Bruno Ubertini” (IZSLER), Gariga Podenzano, 29027 Piacenza, Italy; matteo.ricchi@izsler.it; 4 Dipartimento di Scienze Veterinarie, Università degli Studi di Perugia, 06126 Perugia, Italy; katia.cappelli@unipg.it

**Keywords:** paratuberculosis, *Marchigiana* beef cattle, faecal miRNA, non-invasive biomarkers, TaqMan RT-qPCR, gene expression profiling, host–pathogen interaction

## Abstract

MicroRNAs (miRNAs) are small non-coding RNAs that play a crucial role in the post-transcriptional regulation of gene expression. Since miRNAs modulate host immune responses, they represent promising molecular biomarkers of paratuberculosis (PTB), particularly during early or subclinical stages, when conventional diagnostic tests may lack sensitivity. In this context, faecal miRNAs could provide valuable insights into intestinal immune responses and mucosal damage associated with *Mycobacterium avium* subsp. *paratuberculosis* (MAP) infection. Although miRNAs have been extensively investigated in serum, blood, and tissues, their detection and characterization in bovine faeces remain poorly explored. The aim of this study was to evaluate the expression profiles of selected candidate faecal miRNAs in *Marchigiana* beef cattle naturally exposed to MAP and to assess their association with different infection phenotypes. Thirty-four cows were classified into three phenotypic groups: healthy exposed, MAP-infected, and PTB-affected based on longitudinal diagnostic records including interferon-γ assay, serological testing (ELISA), and faecal qPCR. Five candidate miRNAs were selected from previous studies and quantified in faecal samples by RT-qPCR. Four of the five selected miRNAs were consistently detected across samples. *Bta-miR-92a* was significantly downregulated in both MAP-infected and PTB-affected animals compared with healthy cattle, suggesting early modulation during MAP infection. *Bta-miR-223* was significantly upregulated in PTB-affected animals compared with both healthy and MAP-infected groups, consistent with its established role in the regulation of intestinal inflammation. The ortholog of *hsa-miR-501-5p* was significantly upregulated in MAP-infected cattle, potentially reflecting early host–pathogen interactions at the intestinal mucosal level, while *bta-miR-24-3p* showed no significant differences among groups. Overall, these findings support the feasibility of faecal miRNA analysis as a complementary non-invasive molecular approach to support traditional diagnostic tests for PTB, especially during the early and subclinical stages of MAP infection.

## 1. Introduction

*Mycobacterium avium* subsp. *paratuberculosis* (MAP) is the causal agent of paratuberculosis (PTB), also named Johne’s disease (JD). PTB is globally distributed and affects a wide range of domestic ruminants, including cattle, sheep and goats, as well as wild species [1]. PTB is a chronic granulomatous inflammatory enteritis, transmitted mainly through the faecal–oral route, with significant economic losses in the livestock sector [2,3,4,5]. Clinical onset of the disease typically occurs after a prolonged incubation period, reflecting the slow progression of MAP infection in cattle [5,6,7]. Furthermore, the potential role of MAP as a zoonotic agent, particularly related to Crohn’s Disease (CD) and to other inflammatory or autoimmune human disorders, is still under investigation [8,9,10,11,12].

Depending on the stage of infection, MAP may lead to a range of clinical manifestations, such as progressive wasting, chronic weight loss, intermittent diarrhoea, and, in advanced cases, death. Infected animals may shed MAP through faeces, contributing to environmental contamination, as the pathogen can survive for prolonged periods outside the host [13,14,15]. Moreover, MAP has been detected in animal-derived food products, including milk, dairy products, and meat, as it has been isolated not only from milk and cheeses but also from muscle tissues of infected cattle, raising concerns about potential foodborne exposure [16,17,18,19].

Current diagnostic approaches, including serological assays, faecal culture, and PCR-based methods, show important limitations, particularly during the early and subclinical stages of infection. These limitations are mainly related to the long incubation period of the disease, intermittent and low-level bacterial shedding, and the delayed development of a detectable humoral immune response. Although circulating antibodies become detectable by ELISA as the infection progresses, they do not confer protective immunity [20]. Overall, due to these diagnostic constraints, culture-based methods are still often regarded as reference tests for MAP detection, despite their low sensitivity and long turnaround time, while faecal PCR-based assays are increasingly used as alternative and, in some cases, complementary tools for direct MAP detection [21,22,23].

Importantly, it should be considered that sub-clinically infected animals play a major role in disease transmission and environmental contamination, since they may intermittently shed MAP in faeces despite the absence of clinical manifestations, thereby representing a critical challenge for effective disease control [24].

In order to investigate early immune responses to MAP infection, diagnostic approaches targeting cell-mediated immunity (CMI), such as interferon gamma (IFN-γ) release assays (IGRAs), have been developed [25]. IFN-γ-based tests measure antigen-specific T-cell responses and may provide supportive information for identifying animals in early or subclinical stages of MAP infection, before the development of detectable humoral response and MAP faecal shedding [20]. Although IFN-γ assays have recognized limitations, including possible cross-reactivity with other mycobacteria [26] and performance variability related to disease stage [20], age and infectious dose at exposure [27], they remain among the few available tools capable of supporting the investigation of early cell-mediated immune responses associated with MAP exposure or infection [20,25,26,27].

The complex pathological mechanisms of PTB suggest the need for additional biomarkers to improve diagnostic accuracy and to support prognostic stratification [3,6,26].

In this context, microRNAs (miRNAs) are stable and biologically informative regulators of host immune and inflammatory responses and have emerged as promising molecular biomarkers to enhance the detection of MAP infection, particularly during early or subclinical stages when conventional diagnostic tests may lack sensitivity [28,29,30,31,32,33].

MiRNAs are a class of conserved, endogenous, small non-coding RNAs, approximately 18–25 nucleotides in length, that regulate gene expression at the post-transcriptional level by inhibiting mRNA translation or promoting target mRNA degradation [34]. Owing to their stability, tissue specificity, and involvement in key regulatory pathways, miRNAs have been widely explored in human medicine as diagnostic and prognostic biomarkers, as well as potential therapeutic targets [35]. Altered miRNA expression has been associated with a wide range of pathological conditions, including inflammatory, immune-mediated, and neoplastic diseases, reflecting their central role in the regulation of host immune responses and cellular homeostasis [36,37,38,39]. With the advent of high-throughput omics technologies, the role of miRNAs has also been extensively explored in animal husbandry, including livestock productivity, welfare, and diseases susceptibility [40,41,42]. In particular, the immunological relevance of circulating miRNAs in body fluids such as whole blood, plasma, and serum has been demonstrated in several animal diseases [43,44,45,46], including PTB [29,30,31,47,48].

The potential application of faecal miRNAs as molecular biomarkers has been recently investigated in human medicine for the screening and diagnosis of intestinal pathologies, including inflammatory bowel disease and colorectal cancer [49,50,51]. Conversely, in veterinary medicine, investigations of faecal miRNAs remain limited, despite their potential diagnostic and prognostic value for several animal diseases, including PTB in cattle. To date, the only study specifically addressing bovine faecal miRNAs was conducted by Shaughnessy et al. [33], in both beef (Simmental) and dairy (Holstein–Friesian) cattle.

Previous investigations have identified distinct genetic profiles associated with resistance and susceptibility to PTB in *Marchigiana* cattle, an Italian native beef breed, highlighting the suitability of this breed as a model for studying host–pathogen interactions in MAP infection [52]. In this context, faecal miRNA expression is expected to vary according to the stage of MAP infection, potentially reflecting the host immune response and genetic background.

The aim of this study was to evaluate the faecal expression of candidate miRNAs in *Marchigiana* cattle naturally exposed to MAP within a longitudinally monitored herd, and to assess their association with phenotypes related to different stages of MAP infection.

## 2. Results

### 2.1. MAP Infectious Status and Animal Phenotyping Outcomes

The MAP infectious status and animal phenotypes were defined using the combined results of the IFN-γ release assay, ELISA for anti-MAP antibody detection, and qPCR for direct detection of MAP in faeces. Group allocation was based on the most recent test results within the context of longitudinal herd monitoring, and phenotypic classifications remained stable over time in most animals.

The IFN-γ assay was performed using avian PPD (derived from *Mycobacterium avium*) and Johnin PPD (derived from *Mycobacterium avium* subsp. *paratuberculosis*), as previously described [20,53], to evaluate early cell-mediated immune responses to MAP.

The three phenotypic groups identified through this approach are reported in Supplementary Table S1. Additional longitudinal diagnostic records available for enrolled animals are summarized in Supplementary Table S2.

In detail, ten animals were classified as healthy but exposed, based on negative results in all the three diagnostic tests. Sixteen animals, negative by ELISA and faecal qPCR but showing IFN-γ reactivity, were operationally categorized as MAP-infected within the context of longitudinal herd surveillance. Conversely, eight animals were classified as PTB-affected, regardless of the IFN-γ test results; all were positive to faecal qPCR assay and five of them were also seropositive by ELISA.

### 2.2. Assay Optimization: miRNA Extraction and RT-qPCR Kit Performance Evaluation

The outcomes of the extraction method and assay optimization are summarized in Supplementary Table S3.

Among the tested workflows, the RNeasy PowerFecal Pro Kit (Qiagen^®^) consistently yielded the highest miRNA concentrations, optimal purity ratios, and concordant total RNA measurements across different quantification platforms, indicating efficient removal of PCR inhibitors typically present in faecal matrices.

In contrast, the Power Microbiome kit (Qiagen^®^), although previously applied in exploratory faecal miRNA studies, resulted in markedly lower RNA recovery and suboptimal purity values. The miRNeasy kit (Qiagen^®^) provided moderate miRNA yields but reduced RNA quality, likely due to incomplete inhibitor removal. Based on these combined qualitative and quantitative criteria, RNeasy PowerFecal Pro Kit (Qiagen^®^) was selected as the most accurate and reliable extraction method for downstream analyses.

Different input RNA amounts were tested during the optimization of the two-step TaqMan™ MicroRNA Assay, resulting in highly comparable and reproducible Cq values for both the endogenous target miRNA (*bta-miR-92a*) and the synthetic spike-in control. These results indicate consistent assay sensitivity and robustness, regardless of the input normalization strategy. Moreover, the inclusion of the spike-in control allowed effective monitoring of extraction and amplification efficiency, supporting its suitability as a quality control tool in faecal miRNA analyses.

The optimized workflow provided a reproducible pipeline for faecal miRNA extraction and RT-qPCR quantification, with effective reduction in matrix-associated inhibition and reliable miRNA detection.

### 2.3. miRNA Expression Analysis Outcomes

Four of the five candidate miRNAs were consistently amplified by RT-qPCR across all 34 faecal samples, including healthy cattle (*n* = 10), MAP-infected (IFN-γ-reactive) animals (*n* = 16), and PTB-affected animals (*n* = 8).

Among the reliably detected miRNAs, *bta-miR-223* and *bta-miR-92a* showed the lowest mean Cq values, indicating a comparatively higher abundance in bovine faeces, in agreement with previous observations [32].

*Bta-miR-24-3p* displayed similar mean Cq values across healthy cattle (31.96 Cq), MAP-infected/IFN-γ-reactive animals (31.52 Cq), and PTB-affected cows (31.35 Cq), suggesting relatively stable expression levels among infection phenotypes.

**Figure 1:**
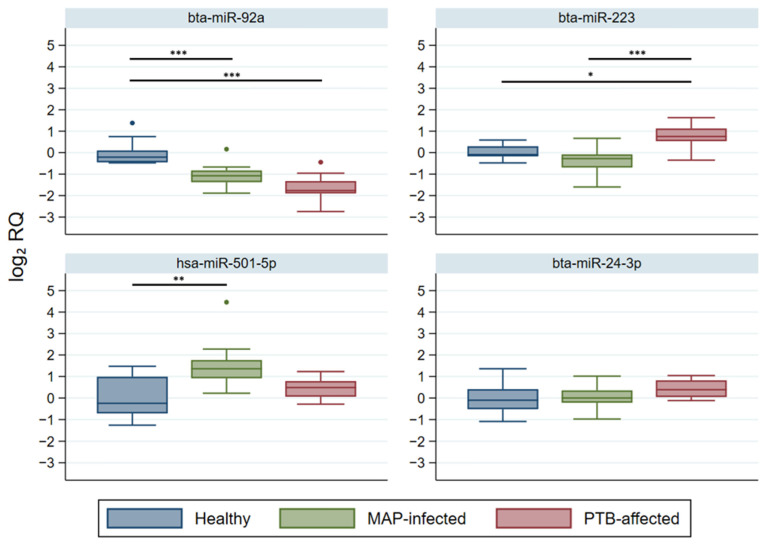
Differential expression of faecal miRNAs across the three phenotypic groups: ELISA negative, qPCR negative, and γ-IFN–negative animals (Healthy exposed, *n* = 10); ELISA and/or qPCR–positive animals (PTB-affected, *n* = 8); and γ-IFN–reactive but ELISA- and qPCR–negative animals (MAP-infected, *n* = 16). The *y*-axis represents relative quantification (RQ = 2^−ΔΔCq^) in log_2_ scale. *bta-miR-92a* was significantly downregulated in both PTB-affected and MAP-infected animals compared with healthy ones. *bta-miR-223* was upregulated in PTB-affected animals compared with healthy ones, and even more markedly compared with MAP-infected animals. *hsa-miR-501-5p* ortholog was upregulated in MAP-infected animals compared with healthy cattle. *bta-miR-24-3p* showed no significant differences among groups. Differences were assessed by one-way ANOVA followed by Bonferroni post hoc test. Boxplots show statistical significance levels: *p* < 0.05 (*); *p* ≤ 0.001 (**); *p* ≤ 0.0001 (***).

As illustrated in Figure 1 and summarized in Table 1, differential expression analysis revealed statistically significant differences among groups based on relative quantification (RQ) for three miRNAs (*bta-miR-92a*, *bta-miR-223*, and the ortholog of *hsa-miR-501-5p*). In contrast, *bta-miR-24-3p* showed only a non-significant trend toward increased expression in PTB-affected animals compared with healthy and MAP-infected groups (*p* > 0.05).

The ortholog of *hsa-miR-501-5p* was significantly upregulated in MAP-infected animals compared with healthy cattle (*p* ≤ 0.001), while only a non-significant overexpression trend was observed when compared with PTB-affected animals. RQ values were generally uniformly distributed within each group, with the presence of a single outlier.

*Bta-miR-223* was significantly upregulated in PTB-affected animals compared with healthy cattle (*p* < 0.05), with an even more pronounced difference when compared with MAP-infected animals (*p* ≤ 0.0001).

Conversely, *bta-miR-92a* showed significantly higher expression levels in healthy animals compared with both MAP-infected and PTB-affected groups (*p* ≤ 0.0001). RQ values were uniformly distributed across the three phenotypic groups, each showing the presence of one outlier.

Finally, *bta-miR-658* was inconsistently detected, with amplification occurring at late cycles (Cq > 37) and mainly in healthy animals, indicating, under the experimental conditions applied, a very low abundance of this miRNA in bovine faecal samples.

## 3. Discussion

This study provides a methodological and biological evaluation of faecal miRNAs as potential non-invasive biomarkers in bovine PTB, focusing on longitudinally defined infection phenotypes and an extensively optimized analytical workflow. Rather than aiming at a discovery-driven approach, the study was designed to assess selected candidate miRNAs previously associated with MAP infection or intestinal inflammatory processes, using a targeted RT-qPCR strategy applicable in the future, after extensive validation, to routine diagnostic matrices.

In the absence of universally stable or validated endogenous miRNA controls in bovine faecal samples, we adopted a data-driven normalization strategy based on the mean Cq of the measured miRNA panel. While global mean normalization is typically recommended for large-scale profiling studies, this approach has also been appropriately applied in contexts where no reliable reference miRNAs are available. Although normalization based on the mean Cq of a limited candidate miRNA panel may potentially introduce bias, this approach was adopted due to the current lack of validated endogenous reference miRNAs for bovine faecal samples and in accordance with previous exploratory studies on bovine faecal miRNAs [33].

Previous investigations have explored the bovine faecal miRNome, most notably the study by Shaughnessy et al. [33], which characterized faecal miRNA expression using NanoString nCounter technology and identified three miRNAs differentially expressed between PTB-positive and healthy cattle. However, that study relied on broad clinical classifications without distinguishing between subclinical MAP infection and overt PTB; therefore, direct comparisons with the present work should be made with caution, as it focused on MAP-infected and PTB-affected cattle without overt clinical signs.

An additional study by Gupta et al. [31] applied NanoString nCounter technology to serum samples from cattle affected by PTB, identified four candidate miRNAs significantly differentially expressed between groups, although different from those detected in faecal matrices. Similarly, Malvisi et al. [30], using miRNA-seq on whole blood, identified nine differentially expressed miRNAs between negative and MAP-infected cattle. These findings, obtained from different biological matrices such as serum and whole blood, highlight the strong influence of the biological compartment analysed on the miRNA signature associated with MAP exposure or infection.

In line with this evidence, recent integrative transcriptomic studies by Badia-Bringué and Alonso-Hearn [54] have highlighted that miRNAs, together with other post-transcriptional regulatory mechanisms, contribute to the modulation of host immune responses throughout the progression of MAP infection, from early subclinical phases to more advanced disease stages, reinforcing their potential role as stage-associated biomarkers, in line with approaches based on phenotypic stratification.

In the present study, the stratification of animals based on IFN-γ responsiveness, in the absence or presence of conventional diagnostic positivity, represents a potentially informative approach for investigating early host immune responses associated with MAP exposure or infection [27,52]. Although miRNA expression was assessed at a single sampling timepoint, animal phenotypes were defined through repeated diagnostic testing over long-term follow-up, thereby increasing confidence in group assignment.

Although IFN-γ reactivity alone cannot unequivocally confirm MAP infection, animals classified as MAP-infected in the present study originated from a PTB-positive herd under long-term surveillance and were characterized through the integration of longitudinal immunological, serological, molecular and epidemiological information. Within this framework, IFN-γ-reactive animals were interpreted as biologically compatible with early or controlled stages of MAP infection, characterized by an active cell-mediated immune response rather than overt pathology [20,25,27,52].

Importantly, none of the PTB-affected animals included in this study showed clinical signs such as diarrhoea or cachexia, indicating that the investigated phenotypes do not represent extreme clinical conditions, but rather different stages along the spectrum of MAP infection and disease progression.

Consistent with previous reports, bovine faeces were confirmed to contain a stable and quantifiable repertoire of miRNAs suitable for downstream molecular analyses. Four out of five candidate miRNAs were consistently detected across all phenotypic groups, whereas *bta-miR-658* showed sporadic amplification at very late Cq values, indicating negligible abundance.

The low Cq values observed for *bta-miR-223* and *bta-miR-92a* confirm their high faecal abundance and technical robustness, a prerequisite for biomarker applicability. However, interpretation of differential expression patterns requires careful consideration of animal stratification and disease stage. In this study, *bta-miR-92a* was significantly downregulated in both MAP-infected and PTB-affected animals compared with healthy cattle, suggesting that its modulation may occur early during MAP infection and persists with disease progression. Given the well-established involvement of *miR-92a* in epithelial homeostasis and intestinal barrier integrity [55], these results support its association with intestinal mucosal alterations rather than with MAP burden per se. In cattle, circulating *miR-92a* has also been reported as downregulated during MAP infection, suggesting a broader host response and possible systemic immune modulation [30]. However, direct comparisons with previous studies [33] should be interpreted with caution due to differences in phenotype definition, biological matrix, and cohort composition.

*Bta-miR-223* was significantly upregulated in PTB-affected animals compared with both healthy and MAP-infected groups, showing a clear separation from healthy cattle and an even more marked difference compared with MAP-infected animals. This finding is consistent with evidence from human inflammatory bowel diseases, including CD, where *miR-223* expression levels in serum, intestinal tissue, and faeces of CD patients were significantly higher than those observed in healthy controls [56]. Moreover, *miR-223* has been widely associated with intestinal inflammation and myeloid cell activation [37,57,58].

Johne’s disease and Crohn’s disease have been historically associated for more than a century [10], mainly due to similarities in anatomopathological features and, in advanced stages, partially overlapping clinical manifestations [8,9,12,59]. Nevertheless, despite extensive investigation, the potential role of MAP in Crohn’s disease remains debated and a definitive causal association has not been conclusively established. However, recurring molecular and immunological analogies between PTB and chronic inflammatory disorders continue to be reported [60]. In this context, the significant upregulation of *miR-223* observed in PTB-affected cattle, similarly to findings reported in CD patients [56], supports the involvement of *miR-223* in intestinal inflammatory processes and may reflect shared inflammatory pathways associated with chronic intestinal inflammation.

Overall, these findings suggest that *bta-miR-223* reflects inflammation patterns associated with disease progression rather than with early stages of MAP infection.

A distinctive feature emerging from this study, related to the phenotype herein considered, namely animals reactive exclusively to the IFN-γ test, is the upregulation of an ortholog of *hsa-miR-501-5p* in MAP-infected animals, which was significant only when compared with healthy cattle. This pattern suggests that this miRNA may be associated with early host–pathogen interactions and immune regulatory mechanisms during the subclinical phase of MAP infection, in which IFN-γ-reactive animals have not yet developed overt disease but exhibit immunological evidence of infection. In this context, the observed modulation of the ortholog of *hsa-miR-501-5p*, together with IFN-γ responsiveness, may help distinguish these animals from healthy cattle. Further validation in larger independent cohorts is required to confirm this finding.

An additional strength of this work lies in the use of faecal samples, a matrix already routinely collected for MAP diagnosis through PCR and culture-based methods. The possibility of extracting miRNAs from the same biological material opens new perspectives for integrated molecular analyses, potentially enabling parallel investigation of pathogen detection and host response (miRNAs) without additional sampling. If robust faecal miRNA biomarkers are validated, this approach could enhance the informational value of existing diagnostic workflows.

Given the exploratory nature of the study and the relatively limited sample size, the present study was not designed as a formal diagnostic validation study and therefore did not include receiver operating characteristic (ROC) analyses or predictive performance estimation. Future large-scale validation studies will be necessary to further assess the diagnostic performance and predictive applicability of the identified candidate miRNAs. Nevertheless, the observed miRNA expression patterns support the potential utility of faecal miRNAs as biologically informative non-invasive molecular indicators associated with different phases of MAP host interaction.

Overall, these findings support the feasibility of faecal miRNA analysis as a complementary tool for characterizing host response across different stages of MAP infection. The identification of consistent expression patterns for miRNAs previously described in other cattle breeds, mainly dairy breeds such as Holstein–Friesian, within an Italian beef breed further supports the biological robustness and potential crossbreed relevance of these candidate biomarkers.

Nevertheless, the inclusion of animals from a single longitudinally monitored herd may limit the generalizability of the findings and warrants further investigations in larger and independent populations to better define their diagnostic and prognostic utility, particularly in relation to infection control and host resilience.

Future validation studies on a larger cohort of animals involving multiple herds, different breeds, and longitudinal sampling conditions may help identify stable endogenous reference miRNAs or other invariant small RNA controls suitable for standardized normalization strategies in bovine faecal miRNA diagnostic workflows.

## 4. Materials and Methods

### 4.1. Ethical Statements

*Marchigiana* breed cattle included in this study were subjected to diagnostic analyses performed by *Istituto Zooprofilattico Sperimentale dell’Umbria e delle Marche “Togo Rosati”* (IZSUM, Perugia, Italy). Samples were collected during official state prophylaxis and routine PTB control activities, in accordance with the Italian National Guidelines, State—Regions Agreement of 30 November 2022 [61]. No additional procedures were performed for research purposes. In accordance with the Italian Legislative Decree 26/2014 implementing Directive 2010/63/EU [62], the study did not fall within the scope of animal experimentation. The Institutional Animal Welfare Body (OPBA) of IZSUM confirmed that ethical approval was not required. Informed consent was obtained from the animal owner.

### 4.2. MAP Infectious Status and Animal Phenotyping

Thirty-four *Marchigiana* cows were selected from a single herd officially free from bovine tuberculosis (bTB) but with a previous history of PTB positivity. As part of routine PTB surveillance, the herd had been monitored longitudinally, with most enrolled animals followed for up to six years. Younger animals entered the monitoring program later according to herd replacement dynamics. The herd included several long-lived animals belonging to the original breeding nucleus and monitored over multiple years. Phenotypic classification was based on the most recent diagnostic results, integrated with previous serological, molecular, and IFN-γ records collected during the monitoring period. This integrated longitudinal approach was consistent with previously proposed conceptual frameworks for PTB case-definition and phenotypic stratification in longitudinal studies by Whittington et al. [63]. The detailed phenotypic classification of enrolled animals is reported in Supplementary Table S1. Longitudinal diagnostic records collected during herd surveillance are additionally summarized in Supplementary Table S2.

Blood and faecal samples were collected during routine herd health activities and processed shortly after collection at the IZSUM laboratory. Faecal samples were stored at −80 °C until analysis.

MAP infectious status and animal phenotypes were determined using an IFN-γ assay to evaluate the early cell-mediated immune response, ELISA tests for anti-MAP antibody detection, and qPCR for direct detection of MAP in faeces. The term “MAP-infected” was operationally used to identify animals showing evidence compatible with MAP infection within the epidemiological context of a PTB-positive herd, including IFN-γ reactivity and/or longitudinal immunological evidence in the absence of overt disease.

Specifically, cell-mediated immunity was assessed on heparinised whole blood by measuring IFN-γ production following in vitro lymphocyte stimulation with purified protein derivatives (PPDs) from *Mycobacterium bovis* (bovine PPD) and *Mycobacterium avium* (avian PPD) as well as an experimental Johnin PPD derived from *Mycobacterium avium* subsp. *paratuberculosis* and produced by the IZSUM, as previously described [21,53].

The IFN-γ response was measured using the commercial BOVIGAM™ ELISA kit (Thermo Fisher Scientific, Waltham, MA, USA) according to the manufacturer’s instructions. The entire procedure, with the relative assay interpretative criteria, has been described in detail by Corneli et al. (2021) [20].

Humoral immunity was assessed using two commercial ELISA kits (ID Screen^®^ Paratuberculosis Indirect, IDVet Innovative Diagnostics, Montpellier, France; and IDEXX^®^-Westbrook, ME, USA) following the manufacturers’ protocols.

Direct molecular detection of MAP DNA was performed on faecal samples by quantitative PCR (qPCR) targeting the *IS900* gene, using a validated Fast Mode protocol [64,65].

### 4.3. Candidate miRNA Selection

MiRNA nomenclature follows standard miRBase conventions, where prefixes indicate species origin (e.g., *bta* for *Bos taurus* and *hsa* for *Homo sapiens*). One of our targets refers to bovine sequences identified as orthologs of the corresponding human miRNAs, based on sequence homology and previous annotation studies [33]. Accordingly, throughout the manuscript, these targets are referred to as “orthologs of *hsa-miR*”, whereas the prefix *bta* is used for bovine orthologs already annotated and deposited in miRbase.

Five candidate and representative faecal miRNAs were selected for expression analysis (Table 1). Three targets, *bta-miR-658*, the ortholog of *hsa-miR-501-5p* and *bta-miR-92a*, were included as they were previously reported to be differentially expressed (DE) in bovine faeces from negative and clinically JD-affected cattle [33].

Additionally, *hsa-miR-223* has been proposed as a potential and novel non-invasive biomarker for assessing disease activity in CD patients [56,58]. Therefore, the bovine orthologue *bta-miR-223* was included in this study given the recognized pathological and clinical similarities between CD and JD.

Finally, *bta-miR-24-3p* was included because it showed significantly increased expression levels in MAP-exposed cattle compared with the negative control group in the study of *Malvisi* et al. [30].

An overview of the candidate miRNAs, corresponding TaqMan assays, and sequence conservation between bovine miRNAs and their human orthologs is provided in Supplementary Table S4.

### 4.4. Assay Optimization: miRNA Extraction and RT-qPCR Quantification Kit Evaluation

An extensive optimization workflow was conducted to identify the most suitable extraction and quantification strategy for faecal miRNA analysis in the context of PTB. Outcomes of the comparison among extraction methods are reported in Supplementary Table S3. Two bovine faecal samples, collected from cattle previously classified as healthy and PTB-affected by conventional diagnostic assays, were selected and processed with and without the addition of *ath-miR-159a* from *Arabidopsis thaliana* (Thermo Fisher Scientific) as an exogenous spike-in control. The spike-in control was added at a fixed amount prior to miRNA extraction to monitor technical variability across extraction, reverse transcription and qPCR steps. Three commercially available RNA extraction kits, RNeasy PowerFecal^®^ Pro Kit (Qiagen^®^, Hilden, Germany), RNeasy^®^ Power Microbiome (Qiagen^®^), and miRNeasy^®^ Mini kit (Qiagen^®^), were comparatively evaluated, according to the manufacturer recommendations for stool matrices, with protocol adaptations, including standardized mechanical lysis by TissueLyser (Qiagen^®^), controlled input material, and optimized elution volumes to improve miRNA recovery.

The optimization phase was intentionally designed as a preliminary technical assessment aimed at identifying the most suitable extraction workflow for bovine faecal miRNA profiling prior to the exploratory biomarker analysis. Due to the exploratory nature of this step, optimization was performed on a limited number of representative samples. Once optimized, the selected extraction protocol was consistently applied to all study samples under the same analytical conditions.

RNA extracts were quantified using fluorometric assays (Qubit 4 Fluorometer, Thermo Fisher Scientific), specific for small RNAs (Qubit™ microRNA Assay Kits, Thermo Fisher Scientific) and total RNA (Qubit™ RNA BR Assay Kits, Thermo Fisher Scientific), to accurately estimate miRNA abundance relative to the total RNA content, a critical parameter for normalization and reverse transcription input selection.

Spectrophotometric analysis was additionally performed, using a Biophotometer (Eppendorf^®^, Hamburg, Germany), on representative samples to evaluate RNA purity through A260/280 and A260/230 ratios.

To further optimize miRNA quantification, reverse transcription and amplification were performed using two step TaqMan™ chemistry MicroRNA Assays, (Thermo Fisher Scientific), based on stem-loop RT primers, comparing two input strategies: reactions starting from 10 ng of total RNA or from 5 ng of purified miRNA, as defined by fluorometric quantification.

### 4.5. miRNA Expression Analysis

Raw data were elaborated by means of QuantStudio™ 7 Software (Thermo Fisher Scientific). All qPCR reactions were performed in technical duplicates, and mean Cq values were calculated for each miRNA target prior to downstream analysis. For each miRNA target, relative miRNA abundance and the fold change were quantified using the 2^−ΔΔCq^ method described by Livak and Schmittgen [66], slightly modified as suggested by Shaughnessy et al. [33]. Due to the lack of validated endogenous reference miRNAs in bovine faecal samples, normalization was performed using a data-driven approach. Specifically, a normalization factor was calculated as the mean Cq value of all measured miRNAs for each sample, and individual miRNA expression levels were normalized accordingly [33]. This factor was subtracted from the raw Cq values to achieve ΔCq values. Subsequently, for each group (miRNA and sample) ΔΔCq values were calculated by subtracting the mean ΔCq of the negative group from the corresponding ΔCq values.

ΔΔCq values were then transformed to calculate relative quantification (RQ = 2^−ΔΔCq^) or log_2_-transformed (log_2_RQ), for statistical comparisons between groups.

### 4.6. Statistical Elaboration

Animals were classified into three groups (healthy, MAP-infected/IFN-γ–positive, and PTB-affected), treated as a categorical independent variable. RQ values were log_2_-transformed prior to inferential testing to improve variance stabilization and approximate data normality.

Differences in miRNA expression among groups were evaluated using one-way ANOVA, followed by Bonferroni-adjusted pairwise comparisons to control for multiple testing. Assumptions of normality and homogeneity of variance were assessed prior to ANOVA through graphical inspection of Q-Q plots and residuals vs fitted valuesn. No outliers were excluded from the statistical analyses. Statistical significance was set at α = 0.05. Descriptive statistics and graphical summaries (boxplots) were generated to visualize expression patterns across groups (Figure 1).

All statistical analyses were performed using Stata 16.1 (StataCorp LLC, College Station, TX, USA).

## 5. Conclusions

This study demonstrates the potential applicability of faecal miRNAs as non-invasive molecular markers associated with different stages of *Mycobacterium avium* subsp. *paratuberculosis* infection in cattle. By integrating an analytical workflow with a phenotypic stratification based on longitudinal diagnostic evaluation, the present study highlights selected faecal miRNAs as candidate diagnostic biomarkers associated with different phases of MAP host interaction, while also reflecting immune response and intestinal involvement. The use of faecal samples, already routinely collected for MAP detection, further supports the integration of faecal miRNA analysis into current diagnostic protocols.

## Figures and Tables

**Table 1:** Differential expression of selected faecal miRNAs among PTB-affected, MAP-infected, and healthy *Marchigiana* cattle.

miRNA	PTB-Affected	MAP-Infected	Healthy	ANOVA *p*-Value	Bonferroni post hoc Comparisons		
	Mean log_2_RQ ± SD	Mean log_2_RQ ± SD	Mean log_2_RQ ± SD		PTB-Affected vs. Healthy	MAP-Infected vs. Healthy	PTB-Affected vs. MAP-Infected
*bta-miR-92a*	−1.64 ± 0.69	−1.07 ± 0.49	0 ± 0.61	**0.0000**	**0.000**	**0.000**	0.083
*bta-miR-223*	0.76 ± 0.59	−0.38 ± 0.61	0 ± 0.34	**0.0001**	**0.017**	0.275	**0.000**
hsa-miR-501-5p *	0.45 ± 0.50	1.42 ± 1.01	0 ± 0.94	**0.0012**	0.893	**0.001**	0.055
*bta-miR-24-3p*	0.43 ± 0.45	0.03 ± 0.46	0 ± 0.76	0.213	0.354	1.000	0.341

Data are presented as mean log_2_ relative expression (log_2_RQ) ± standard deviation (SD). Log_2_RQ values were calculated using the 2^−ΔΔCq^ method with healthy animals as calibrator (mean = 0). Group differences were evaluated by one-way ANOVA followed by Bonferroni-adjusted pairwise comparisons. Statistical significance was set at *p* < 0.05, and significant values are shown in bold. * Putative bovine ortholog of *hsa-miR-501-5p* identified based on sequence homology; no bovine mature miRNA annotation is currently available in miRBase.

## Data Availability

The data presented in this study are available from the corresponding author upon reasonable request.

## Supplementary Materials

The following supporting information can be downloaded at: https://www.mdpi.com/article/10.3390/ijms27125412/s1.

## Abbreviations

The following abbreviations are used in this manuscript:

MAP	*Mycobacterium avium* subsp. *paratuberculosis*
PTB	Paratuberculosis
JD	Johne’s Disease
miRNA	MicroRNA
RT-qPCR	Reverse Transcription quantitative Polymerase Chain Reaction
qPCR	Quantitative Polymerase Chain Reaction
ELISA	Enzyme-Linked Immunosorbent Assay
IFN-γ	Interferon gamma
CMI	Cell-Mediated Immunity
PPD	Purified Protein Derivative
RQ	Relative Quantification
Cq	Quantification Cycle
RNA	Ribonucleic Acid
mRNA	Messenger Ribonucleic Acid
DE	Differentially Expressed
CD	Crohn’s Disease
ROC	Receiver Operating Characteristic
