# Supplementary material for: Candidate Faecal microRNAs as Non-Invasive Biomarkers for Bovine Paratuberculosis in Marchigiana Beef Cattle
Source: Int J Mol Sci. 2026 Jun 16;27(12):5412. doi: 10.3390/ijms27125412 (PMC13300290; doi:10.3390/ijms27125412)
Supplement: Supplementary file 1 [file ijms-27-05412-s001.zip › ijms-4336179-supplementary.pdf]

International Journal of
Molecular Sciences

Supplementary Materials

Supplementary Table S1. Phenotypic classification of enrolled animals based on serological, molecular, and cell-mediated immune response tests.

**Phenotype**	**Diagnostic criteria**	**n**
Healthy	Negative ELISA, negative faecal qPCR, and negative IFN-γ assay	10
MAP-infected	Negative ELISA, negative faecal qPCR, and reactive IFN-γ assay	16
PTB-affected	Positive faecal qPCR and/or positive ELISA, regardless of IFN-γ result	8

Supplementary Table S2. Longitudinal diagnostic characterization of animals included in the present study. Age refers to the age of animals at the 2023 sampling timepoint used for faecal miRNA profiling. The table summarizes the most representative longitudinal diagnostic records available during the herd surveillance period preceding the 2023 sampling. Animals classified as “IFN-γ-reactive” showed reactivity against one or both purified protein derivatives (PPDs) used in the interferon-gamma release assay despite negative faecal PCR and ELISA results. Av indicates reactivity against *Mycobacterium avium* PPD, whereas JB indicates reactivity against Johnin PPD derived from *Mycobacterium avium* subsp. *paratuberculosis* (MAP). NA indicates unavailable data or animals not sampled during the corresponding year. Values in parentheses for ELISA represent S/P ratios, whereas values in parentheses for faecal PCR represent quantification cycle (Cq) values obtained by *IS900* real-time PCR. According to the validated assay protocol, samples with Cq values >36 were interpreted as negative. Healthy animals were not reported because they consistently tested negative throughout the longitudinal monitoring period.

Due to table formatting, the complete Supplementary Table S2 is presented on the following landscape-oriented page.

International Journal of Molecular Sciences

Animal ID	Age (years)	2019 ELISA	2019 IFN-γ	2020 ELISA (S/P)	2020 IFN-γ	2020 Faecal qPCR (Cq)	2023 ELISA (S/P)	2023 Faecal qPCR (Cq)	2023 IFN-γ	2023 Study Group	Final interpretation
**3**	7	Negative	JB	Negative	JB	Negative	Negative	Negative (38.1)	JB	IFN-γ-reactive	Persistent IFN-γ reactivity
**7**	9	Negative	Av/JB	Negative	Av/JB	Negative	Negative	Negative (37.6)	Av/JB	IFN-γ-reactive	Persistent IFN-γ reactivity
**8**	6	NA	NA	Negative	JB	Negative	Negative	Negative (38.2)	JB	IFN-γ-reactive	Persistent IFN-γ reactivity
**9**	6	NA	NA	Negative	JB	Negative	Negative	Negative (39.4)	JB	IFN-γ-reactive	Persistent IFN-γ reactivity
**12**	19	Negative	JB	Negative	JB	Negative	Negative	Negative (37.5)	JB	IFN-γ-reactive	Persistent IFN-γ reactivity; cow belonging to the original herd nucleus.
**15**	5	NA	NA	Negative	Av/JB	Negative	Negative	Negative (37.0)	Av/JB	IFN-γ-reactive	Persistent IFN-γ reactivity
**23**	4	NA	NA	Negative	JB	Negative	Negative	Negative (39.1)	JB	IFN-γ-reactive	Persistent IFN-γ reactivity
**27**	8	Negative	JB	Negative	JB	Negative	Negative	Negative (38.4)	JB	IFN-γ-reactive	Persistent IFN-γ reactivity
**30**	3	NA	NA	NA	NA	NA	Negative	Negative (38.7)	Av/JB	IFN-γ-reactive	Single-timepoint IFN-γ reactivity
**31**	6	NA	NA	Negative	JB	Negative	Negative	Negative (>40)	JB	IFN-γ-reactive	Persistent IFN-γ reactivity
**34**	2	NA	NA	NA	NA	NA	NA	Negative (39.9)	JB	IFN-γ-reactive	Single-timepoint IFN-γ reactivity
**35**	2	NA	NA	NA	NA	NA	NA	Negative (38.2)	Av/JB	IFN-γ-reactive	Single-timepoint IFN-γ reactivity
**37**	7	Negative	Negative	Negative	JB	Negative	Negative	Negative (>40)	JB	IFN-γ-reactive	Delayed IFN-γ reactivity
**38**	8	Negative	JB	Negative	JB	Negative	Negative	Negative (39.7)	JB	IFN-γ-reactive	Persistent IFN-γ reactivity
**41**	7	Negative	JB	Negative	JB	Negative	Negative	Negative (37.9)	JB	IFN-γ-reactive	Persistent IFN-γ reactivity
**46**	5	NA	NA	Negative	Av	Negative	Negative	Negative (38.8)	Av	IFN-γ-reactive	Persistent IFN-γ reactivity
**16**	7	Negative	JB	Positive (234)	JB	Positive (25)	Positive (234)	Positive (33)	JB	PTB-affected	Longitudinally confirmed PTB
**17**	6	Negative	Av/JB	Positive (240)	Av/JB	Positive (26)	Positive (240)	Positive (26)	Negative	PTB-affected	Longitudinally confirmed PTB
**18**	6	Negative	Av/JB	Positive (253)	Av/JB	Positive (25)	Positive (253)	Positive (25)	Negative	PTB-affected	Longitudinally confirmed PTB
**24**	5	NA	NA	Negative	JB	Negative	Negative	Positive (34)	Negative	PTB-affected	PCR-positive in 2023
**32**	3	NA	NA	NA	NA	NA	Negative	Positive (34.2)	Av/JB	PTB-affected	PCR-positive in 2023
**33**	2	NA	NA	NA	NA	NA	Negative	Positive (36)	Negative	PTB-affected	PCR-positive in 2023
**39**	3	NA	NA	NA	NA	NA	Positive (248)	Positive (32)	JB	PTB-affected	Confirmed PTB (offspring of animal 17)
**45**	3	NA	NA	NA	NA	NA	Positive (227)	Positive (35)	JB	PTB-affected	Confirmed PTB (offspring of animal 18)

International Journal of
Molecular Sciences

Supplementary Table S3. Qualitative and quantitative comparison of miRNA extraction methods using faecal samples from PTB-negative (N) and PTB-positive (P) cattle.

		QUBIT (Thermo Fisher ®)		Biophotometer (Eppendorf®)		
**Extraction kits (Qiagen®)**	**Sample**	**miRNA (ng/µl)**	**RNA (ng/µl)**	**RNA (ng/µl)**	**260/280 ratio**	**260/230 ratio**
**RNeasy Power Fecal**	1N	68	236	252	1.85	1.50
	2N	60	235	250	1.80	1.45
	3P	44	102	105	1.85	1.45
	4P	58	259	120	1.80	1.50
**Power Microbiome**	1N	4	17	17	1.40	0.40
	2N	3	16	25	1.30	0.50
	3P	5	18	28	1.40	0.42
	4P	3	11	23	1.35	0.75
**miRNeasy**	1N	29	92	163	1.40	0.70
	2N	72	103	300	1.30	0.81
	3P	61	95	265	1.35	0.75
	4P	66	96	267	1.30	0.81

International Journal of *Molecular Sciences*

Supplementary Table S4. Integrated overview of selected miRNAs analysed in this study, including species annotation, and sequence comparison between bovine miRNAs and corresponding human orthologs.

miRNA	Species annotation	miRBase accession	Bovine mature sequence (5′→3′)	Human orthologs	Human mature sequence (5′→3′)	Sequence conservation
bta-miR-92a	Bovine	MIMAT0009383	UAUUGCACUUGUCCCGGCCUGU	*hsa-miR-92a-3p*	UAUUGCACUUGUCCCGGCCUGU	Identical
**Bovine ortholog of *hsa-miR-501-5p****	No bovine mature annotation available	Not available	Not annotated (human ortholog sequence adopted for assay design)	*hsa-miR-501-5p*	AAUCCUUUGUCCCUGGGUGAGA	Human TaqMan assay adopted
bta-miR-658	Bovine	MIMAT0009362	GGCGGAGGGAAGCGGGUCCGUUGGU	*hsa-miR-658*	GGCGGAGGGAAGUAGGUCCGUUGGU	Single mismatch
bta-miR-223	Bovine	MIMAT0009270	UGUCAGUUUGUCAAAUACCCCA	*hsa-miR-223-3p*	UGUCAGUUUGUCAAAUACCCCA	Identical
bta-miR-24-3p	Bovine	MIMAT0003840	UGGCUCAGUUCAGCAGGAACAG	*hsa-miR-24-3p*	UGGCUCAGUUCAGCAGGAACAG	Identical

*The orthologous bovine miRNA was identified based on sequence homology with hsa-*miR-501-5p* and analysed using the human TaqMan™ MicroRNA Assay ID 001047_mir/hsa-miR-501-5p (Thermo Fisher Scientific).
